# Mapping the storm: a scoping review of diabetic emergencies in Africa

**DOI:** 10.3389/fcdhc.2026.1822436

**Published:** 2026-05-18

**Authors:** Alexis Harerimana, Gugu Mchunu

**Affiliations:** 1 Faculty of Health Sciences, Durban University of Technology, Durban, KwaZulu-Natal, South Africa; 2 College of Healthcare Sciences, James Cook University, Townsville, QLD, Australia

**Keywords:** acute diabetic complications, Africa, diabetic emergencies, diabetic ketoacidosis, hyperosmolar hyperglycemic state, severe hypoglycemia

## Abstract

**Objective:**

This scoping review aimed to map the evidence on the patterns and burden of diabetic emergencies among people with diabetes in Africa.

**Methods:**

The review followed the Levac et al. framework and the PRISMA-ScR guidelines. A systematic search was conducted across five electronic databases (CINAHL, Embase, MEDLINE, Scopus, and Web of Science), and manual searches in Google and Google Scholar were conducted for studies published between 2015 and 2025. From 1,207 records, 25 studies met the inclusion criteria. Extracted data were analyzed using thematic analysis and descriptive numerical summaries.

**Findings:**

Of the 25 reviewed studies, diabetic ketoacidosis (DKA) was the most commonly reported emergency, appearing in 17 studies, with proportions ranging from 2.0% to 92.6%. This was followed by hyperosmolar hyperglycemic state (HHS), reported in 11 studies (4.5%–50.6%), and hypoglycemia, reported in 9 studies (0.8%–28.3%). Common determinants included infections and intercurrent illness, poor glycemic control, treatment non-adherence or discontinuation, and limited access to ongoing care. Reported in-hospital mortality ranged from 5% to 17.1%, with DKA case fatality reaching 30% in some studies. Median hospital stay generally ranged from 6 to 9 days.

**Conclusion:**

Diabetic emergencies are a major, yet largely preventable, cause of illness and death among people with diabetes in Africa. Conditions such as DKA place a heavy strain on African healthcare systems. To reduce the rates of these avoidable complications, it is essential to improve primary care for early detection and ensure consistent access to insulin and glucose monitoring.

## 1 Introduction

Diabetic emergencies are a major global health challenge and broadly include hyperglycemic crises, such as diabetic ketoacidosis (DKA) and hyperosmolar hyperglycemic state (HHS), as well as hypoglycemic crises, including severe hypoglycemia (1–4). In people with type 1 diabetes (T1DM), hyperglycemic crisis rates range from 44.5 to 82.6 per 1,000 person-years (5). Similarly, McCoy et al. (6) found that the incidence of hyperglycemic emergencies varies by diabetes type, with adjusted rates of 47.8 per 1,000 person-years for T1DM compared to 3.7 per 1,000 person-years for type 2 diabetes (T2DM) (6). The literature shows that DKA is the most prevalent diabetic emergency (1, 5, 7). The 2024 Consensus Report by the American Diabetes Association (ADA) and the European Association for the Study of Diabetes (EASD) documented that DKA hospitalizations have risen by 55% over the last ten years.

Population-based data indicate that hospital admission rates for DKA increased from 7.22 to 9.49 per 100,000 person-years between 2010 and 2018 (8). Younger individuals, particularly those diagnosed with T1DM, are more susceptible to DKA, which is typically precipitated by insulin discontinuation and infections (5, 9–11). In contrast, globally, HHS, representing less than 1% of diabetes-related admissions, carries disproportionate lethality (12). A study by Rosager et al. (12) reported incidence rates of 16.5 and 3.9 per 10,000 person-years for T1DM and T2DM, respectively, with in-hospital mortality of 17% for pure HHS, and 32% of cases presenting without a prior diabetes diagnosis. HHS predominantly affects older adults with T2DM, often complicated by comorbidities such as cardiovascular disease and a prolonged history of diabetes (5, 9, 13). Specifically, Benoit et al. (14) reported that 47.5% of HHS cases occurred in middle-aged adults (aged 45–64 years), with T2DM accounting for 88.1% of these instances. The elderly population, particularly those over 60 years of age, frequently presents with atypical forms of DKA triggered by infections or non-infectious comorbidities, resulting in poorer outcomes compared to younger patients (10).

Diabetic emergency presentations also include severe hypoglycemia, which remains highly prevalent (15, 16). A study by Sherr et al. (15) reported that approximately 20% of T1DM individuals experienced at least one severe hypoglycemic event within 12 months, even with widespread use of advanced diabetes technologies. The iNPHORM prospective study further found that 33.8% of insulin-treated adults in the United States experienced at least one level 3 hypoglycemic event annually—44.2% in T1DM and 30.8% in T2DM—confirming the persistent and disproportionate burden of these emergencies across diverse healthcare settings (16).

Internationally, patterns of diabetic emergencies differ (6, 17–19). In Singapore, admission rates for DKA and HHS rose consistently over a decade, despite improvements in glycemic control, particularly among individuals with chronic kidney disease and prolonged diabetes duration (19). In the United States, inequities in healthcare access and broader social determinants of health exacerbate the burden of diabetic emergencies (6, 18). Diabetic emergencies pose a disproportionate threat in low- and middle-income countries (LMICs), where DKA episodes range from 3.8% to 73.4%, HHS from 0.9% to 58%, and severe hypoglycemia from 3.3% to 64.7% annually (4). Socioeconomic status serves as a critical determinant, as lower income and inadequate glycemic control significantly elevate the risk of hyperglycemic crises in both type 1 and type 2 diabetes (6).

The clinical consequences of diabetic emergencies extend beyond the acute episode. The risk of mortality is heightened when DKA or HHS co-occurs with cardiovascular events (20, 21). In patients hospitalized for ST-elevation myocardial infarction, concurrent DKA increased the adjusted odds of in-hospital mortality by 2.3-fold and acute renal failure by nearly 5-fold, while HHS similarly exacerbated hemodynamic instability and cardiogenic shock (20). The DKA–HHS overlap syndrome, affecting up to one-third of patients presenting with hyperglycemic crises, has a mortality rate of approximately 8%, exceeding that of isolated DKA (3%) or HHS (5%), with compounded risks of cerebral edema, deep vein thrombosis, and acute kidney injury (21). In-hospital DKA mortality among adults is approximately 3.9%, with ICU admission required in over 55% of adolescent admissions and one-year readmission rates of 22% (8).

The economic burden of diabetic emergencies is substantial and inequitable. In the United Arab Emirates, the mean hospitalization cost per DKA admission was USD 12,274, with ICU utilization exceeding 61% of all admissions. Notably, the cost of a single preventable DKA admission equaled 27 years of insulin therapy for one patient (22). In the United States, annual DKA-related hospitalization costs have reached USD 6.6 billion, with indirect costs from lost productivity adding a further 30–40% to direct medical expenses (22). In resource-limited settings such as Ethiopia, DKA recurrence remains persistently high, driven by medication non-adherence and infection, with comorbidity independently increasing recurrence risk by 1.58-fold, highlighting systemic gaps in outpatient follow-up and access to medications (23).

In Africa, the prevalence of DKA and HHS remains significantly high, reflecting systemic challenges in diabetes management (24–27). A systematic review in Ethiopia found a pooled prevalence of hyperglycemic crises of 45.37%, with DKA accounting for 40.77% and HHS for 8.56%, primarily among patients with T2DM (24). Comparable trends have been documented elsewhere on the continent: in the Ivory Coast, DKA occurred in 30.7% of patients (26), while in Nigeria, DKA accounted for 85% of hyperglycemic emergencies, with an 18% mortality rate, compared to HHS, which constituted 15% but had a higher mortality rate of 35% (27). Regarding hypoglycemia, Ambaye et al. (28) found that 61.6% (207) of the participants reported experiencing hypoglycemia since their diabetes diagnosis. This included 126 (81.6%) individuals with T1DM and 81 (62.8%) with T2DM. These burdens are compounded by factors such as poor glycemic control, treatment non-adherence, and comorbidities, often linked to socioeconomic challenges and inadequate healthcare resources (2, 24, 29).

Despite established diagnosis and management protocols for diabetic emergencies—including DKA, HHS, and severe hypoglycemia—significant gaps exist in the literature, particularly in developing countries and low-resource settings. Notably, high-quality, prospective epidemiological data on incidence, prevalence, and long-term outcomes are scarce, with most evidence derived from retrospective studies, case series, or reviews rather than randomized controlled trials, which limits robust risk prediction and prevention strategies (4). Furthermore, most data originate from high-income settings, limiting applicability to resource-constrained African contexts, where infection rates, undiagnosed diabetes, and medication non-adherence contribute to a high prevalence of diabetic emergencies (25, 30, 31). Single-site studies highlight substantial burdens of diabetic emergencies but reveal a lack of multi-country data on incidence, risk factors, and long-term outcomes across Africa. Thus, this scoping review aimed to map the evidence on the patterns and burden of diabetic emergencies among people with diabetes in Africa.

## 2 Methods

This scoping review was conducted in accordance with the framework established by Levac et al. (32) and adhered to the PRISMA-ScR guidelines (33). The scoping review methodology consisted of the following sequential steps: identifying the research questions, identifying relevant studies, selecting eligible studies, charting the data, and collating, summarizing, and reporting the results (32).

### 2.1 Stage 1: identifying the research question

The research questions aimed to evaluate the burden of diabetic emergencies in Africa by detailing their prevalence and types, identifying key predictors, and assessing clinical outcomes. Establishing the prevalence and distribution of these emergencies provides essential epidemiological evidence for health planning, resource allocation, and the prioritization of preventive strategies. Identifying predictors of diabetic emergencies is critical for understanding the clinical, behavioral, and health system-related factors that elevate risk. Furthermore, assessing the clinical outcomes of these emergencies is essential for evaluating their impact on patient morbidity and mortality, healthcare utilization, and recovery trajectories. The specific research questions were:

- What types and prevalence of diabetic emergencies occur among diabetic patients in Africa?
- What are the predisposing factors to diabetic emergencies among diabetic patients in Africa?
- What are the clinical outcomes of diabetic emergencies among diabetic patients in Africa?

### 2.2 Stage 2: identifying relevant studies

A comprehensive search was performed across five electronic databases: CINAHL, Embase, MEDLINE, Scopus, and Web of Science. Supplementary searches were conducted using Google Scholar and manual screening of the reference lists of included studies to identify additional pertinent literature. The search was limited to studies published between 2015 and 2025 to ensure the inclusion of contemporary data. Truncation (*), quotation marks (“ “), and Boolean operators (AND/OR) were used to capture word variants, retrieve exact multi-word phrases, and systematically combine search concepts, thereby enhancing the sensitivity, precision, and comprehensiveness of the database search.

− S1: “Diabetic ketoacidosis” OR DKA OR “hyperosmolar hyperglyc* state” OR HHS OR hypoglyc?mia OR “hyperglyc* crisis” OR “diabetic emergenc*” OR “acute diabetic complications.”
− S2: “Diabetic patients” OR “people with diabetes” OR “diabetes mellitus” OR “type 1 diabetes” OR “type 2 diabetes.”
− S3: Prevalence OR epidemiolog* OR incidence.
− S4: Africa.
− S5: S1 AND S2 AND S3 AND S4.

### 2.3 Stage 3: selecting eligible studies

Specific eligibility criteria guided the selection process. Studies were required to be conducted in Africa and report on the prevalence, incidence, or patterns of diabetic emergencies. Exclusion criteria encompassed narrative reviews, systematic reviews, and editorials. Studies focusing exclusively on chronic complications or conducted outside Africa were also excluded. Two independent reviewers (AH and GM) screened the titles and abstracts against these criteria, followed by a full-text review of selected articles. Any discrepancies were resolved through discussion and consensus.

**Figure 1:**
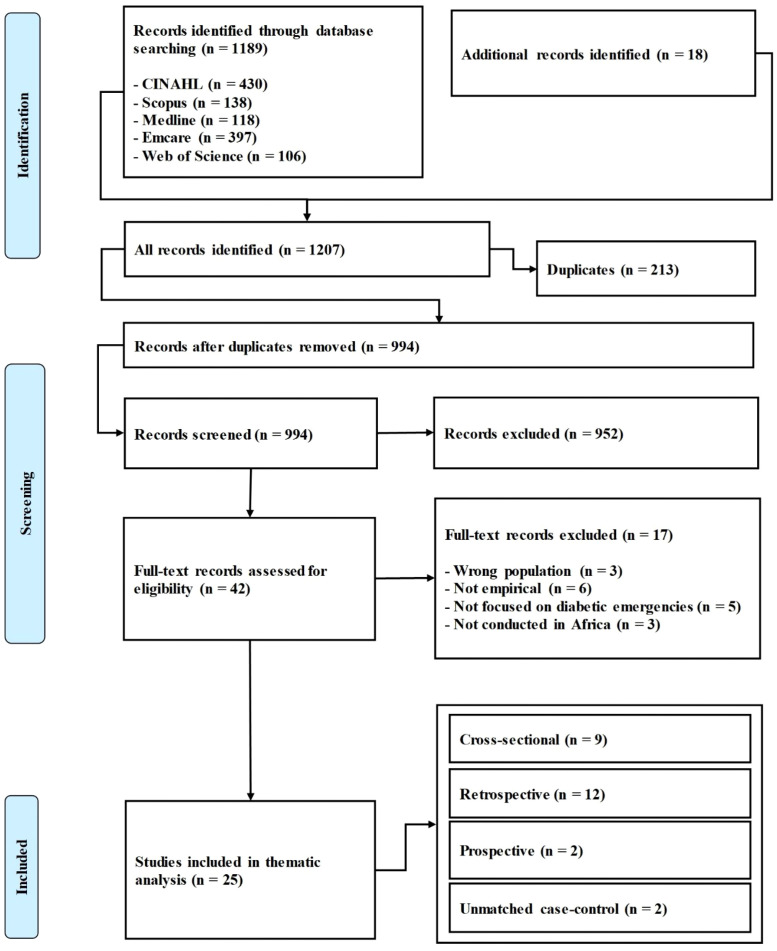
PRISMA flow diagram.

Methodological quality of included studies was assessed using the Mixed Methods Appraisal Tool (MMAT). The MMAT (2018) was selected for its ability to evaluate a diverse range of study designs, including qualitative, randomized controlled, non-randomized quantitative, descriptive quantitative, and mixed-methods studies (34). Each study was categorized into one of the five MMAT design categories and assessed using the tool’s five methodological criteria, with ratings recorded as “Yes”, “No” or “Can’t tell” (34). Although no studies were excluded due to quality concerns, MMAT evaluations helped identify methodological strengths and weaknesses within the evidence base. All retrieved references were organized and managed using EndNote version 21. The study selection process was documented using a PRISMA-ScR flow diagram (**Figure 1**), which included reasons for full-text exclusions.

### 2.4 Stage 4: charting the data

A standardized data-charting form was developed and piloted by the research team. Data were extracted from the included studies regarding the author and year of publication, country, study design, population and sample size, and key findings, including the types and prevalence of diabetic emergencies, their predictors, and clinical outcomes. This iterative process ensured the accurate capture of all variables relevant to the research question.

### 2.5 Stage 5: collating, summarizing, and reporting results

The extracted data were analyzed using descriptive numerical summaries and thematic synthesis. Analysis was conducted using the six-phase thematic analysis framework proposed by Braun and Clarke (35). This structured approach improved analytical rigor, consistency, and reliability throughout all stages. The phased process facilitated focused engagement with each analytical step, minimizing the risk of overlooking important details. In Phase 1, the data were read multiple times to become familiar with them. Phase 2 involved generating initial codes by systematically identifying meaningful features. In Phase 3, related codes were grouped into potential themes. Phase 4 included reviewing and refining these themes to ensure they were coherent with both the coded extracts and the entire dataset. During Phase 5, themes were clearly defined and named to capture their core meaning and scope. The final phase involved synthesizing the analysis into a coherent narrative that directly addressed the research questions. Quantitative and qualitative data were integrated to provide a comprehensive understanding of the evidence, and findings were interpreted in line with the review objectives. The results of the scoping review were reported in accordance with the PRISMA-ScR guidelines (33).

## 3 Results

### 3.1 Study characteristics

The initial database searching yielded 1,189 records, including CINAHL (n = 430), Scopus (n = 138), MEDLINE (n = 118), Embase (n = 397), and Web of Science (n = 106). Additionally, 18 records were identified from other sources, bringing the total to 1,207. After removing 213 duplicates, 994 records remained for title and abstract screening, during which 952 were excluded. The full texts of 42 articles were assessed for eligibility; 17 were excluded for not meeting the review criteria. A total of 25 met the inclusion criteria and were included in data extraction and analysis (**Figure 1**). The majority of the included studies employed retrospective designs (n = 12) (7, 11, 25, 31, 36–43), followed by cross-sectional designs (n = 9) (44–52). A smaller proportion used unmatched case-control designs (n = 2) (24, 53), and two were classified as prospective cohort study designs (30, 54). Ethiopia dominated the geographic distribution, contributing 16 of the 25 studies (24, 25, 31, 37, 38, 40, 43–45, 47–49, 51–54). South Africa contributed four studies (7, 30, 39, 42), while Ghana (36), the Democratic Republic of Congo (46), Nigeria (11), Rwanda (41), and Cameroon (50) each provided one study, adding data from West, Central, and East/Southern Africa. Sample sizes for adult cohorts varied from fewer than 100 in single-center admission studies (30, 39, 54) to 1,600 outpatients with T2DM in Ghana (36). Hyperglycemic crisis cohorts typically included 150–600 patients (38, 40). Pediatric studies, all from Ethiopia, included a retrospective follow-up of 389 children with diabetes (31) and a smaller series of 61 newly diagnosed children with T1DM (43) (**Table 1**).

**Table 1:** Summary of the key results of the study.

Author & year	Country	Title	Population & sample size	Study design	Key findings
Abate et al. (25), 2023	Ethiopia	“Incidence and predictors of hyperglycemic emergencies among adult diabetic patients in Bahir Dar city public hospitals, Northwest Ethiopia, 2021: A multicenter retrospective follow-up study.”	Adult DM patients (n = 453)	Retrospective	− Overall, 32.45% (147/453) experienced ≥1 hyperglycemic emergency. The incidence was 14.6 per 100 person-years. DKA accounted for most events (12.5 per 100 person-years), occurring more frequently in T1DM (35.6%) than T2DM (6.3%), while HHS was less common (2.1 per 100 person-years), mainly among T2DM (2.4%). − Higher risk was associated with T1DM (AHR 2.75), acute illness (AHR 2.99), comorbidities (AHR 2.36), poor glycemic control (AHR 3.47), non-adherence (AHR 1.85), infrequent follow-up (AHR 1.79), and lack of insurance (AHR 1.63); ≥3 years’ duration was protective (AHR 0.33). − Median free survival was 53.85 months.
Abejew et al. (44), 2015	Ethiopia	“Diabetic complications among adult diabetic patients of a tertiary hospital in Northeast Ethiopia.”	Adult DM patients (n = 216)	Cross-sectional	− Diabetes-related complications affected 59.7% (129/216) of patients. − Acute complications accounted for 28.2%, with DKA as the predominant emergency (68.3%), followed by hypoglycemia (28.3%) and HHS (7.9%). − Acute events occurred in both diabetes types, with a higher overall complication rate among T2DM. − Complications were significantly associated with age (p = 0.048), type of diabetes (p < 0.001), medication regimen (p = 0.001), and specific antidiabetic drugs (p < 0.001). Adherence, attitudes, knowledge, average blood glucose, and family history were not significantly associated. − Chronic complications were more frequent than acute (58.8% vs 41.2%), with hypertension (43.3%), visual disturbances (28.9%), neuropathy (14.4%), foot ulcers (4.4%), nephropathy (2.2%), and impotence (2.2%) most common.
Annani-Akollor et al. (36), 2019	Ghana	“Predominant complications of T2DM in Kumasi: A 4-year retrospective cross-sectional study at a teaching hospital in Ghana.”	Adult T2DM patients (n = 1,600)	Retrospective	− Overall, 59.0% of patients experienced at least one diabetes-related complication. − Acute hyperglycemic emergencies were uncommon, with DKA (2.0%) and hypoglycemia (0.8%). − Macrovascular complications affected 31.8%, while microvascular complications occurred in 35.3%. Neuropathy was the most common microvascular complication (20.8%), followed by nephropathy (12.5%) and retinopathy (6.5%). − Employment status was protective, whereas longer diabetes duration increased risk, particularly 5–10 years (aOR 1.55) and >10 years (aOR 2.76). − Most patients were older adults and had ≥5 years’ diabetes duration.
Bedaso et al. (37), 2019	Ethiopia	“Diabetic ketoacidosis among adult patients with diabetes mellitus admitted to the emergency unit of Hawassa University Comprehensive Specialized Hospital.”	Adult DM patients (n = 195)	Retrospective	− DKA affected 40.0% (78/195) of patients and accounted for 77% of acute complications, followed by hypoglycemia (14%) and HHS (9%). − DKA occurred in both diabetes types but was more frequent in T1DM (28.2%) than T2DM (11.8%). Notably, 53.9% of newly diagnosed patients presented with DKA. − Younger age was protective against DKA (15–24 years: OR 0.21, 95% CI 0.072–0.60; 25–34 years: OR 0.22, 95% CI 0.087–0.54), whereas comorbid hypertension increased risk (AOR 2.78, 95% CI 1.095–7.074; p < 0.05). − Patients commonly presented with classic hyperglycemic symptoms and weight loss, with a high rate of chronic complications, including diabetic foot ulcers (60%), retinopathy (27%), and nephropathy (13%). The cohort was predominantly male (64.1%), and 34.4% were newly diagnosed at presentation.
Birhanu et al. (45), 2025	Ethiopia	“Prevalence and associated factors of diabetic ketoacidosis among patients with diabetes mellitus at the University of Gondar Comprehensive and Specialized Referral Hospital, Northwest, Ethiopia.”	DM patients (n = 405)	Cross-sectional	− DKA prevalence was 8.6% (35/405; 95% CI 6.0–11.0), defined by fasting blood glucose ≥250 mg/dL, urine ketones ≥2+, urine pH <7, and serum bicarbonate <15 mEq/L. − Most cases occurred in T1DM (71.4%), though 28.6% were in T2DM. The highest frequency was among those aged 20–29 years (48.6%). − Increased odds of DKA were associated with age 20–29 years (AOR 2.26), unemployment (AOR 2.58), infection (AOR 2.82), and T1DM (AOR 3.11).
Cedrick et al. (46), 2021	Democratic Republic of Congo	“Prevalence and determinants of poor glycemic control amongst patients with diabetes followed at Vanga Evangelical Hospital, Democratic Republic of the Congo.”	DM patients (n = 300)	Cross-sectional	− Poor glycemic control was highly prevalent, with 78% of patients having uncontrolled HbA1c (≥7.0%). This burden affected both T1DM (37.7%) and T2DM (62.3%), indicating a substantial risk state for hyperglycemic emergencies. − Increased risk was independently associated with tobacco use (aOR 2.01), comorbidities (aOR 2.86), infection (aOR 2.74), missed clinic appointments (aOR 2.59), and treatment non-adherence (aOR 4.09). − More than three-quarters of patients with diabetes receiving treatment had poor glycemic control, and poor adherence accounted for 94.4% of hyperglycemia, followed by infections at 21.4%.
Desse et al. (38), 2015	Ethiopia	“Predictors and treatment outcome of hyperglycemic emergencies at Jimma University Specialized Hospital, southwest Ethiopia.”	DM patients with HEs (n = 163)	Retrospective	− Severe hyperglycemic emergencies were common, with DKA accounting for 92.6% (151/163) and HHS for 7.4%. Most patients had T1DM (64%). − Presentations were marked by hyperglycemia and ketonuria, with altered consciousness in 19.6% (GCS <15) and hypotension in 4.9%. − Infections were the leading precipitant (59%), followed by treatment non- adherence (32.3%) and newly diagnosed diabetes (23.6%). − Independent predictors included serum creatinine >1.2 mg/dL (AOR 5.86), sepsis (AOR 9.83), and comorbidities (AOR 15.26). UTIs were the most common infectious trigger (64.2%), and medication discontinuation accounted for the majority of non- adherence (86.5%). − Median stay was 6 days, with 9.8% in-hospital mortality and frequent complications, including recurrent hyperglycemia (54%), hypoglycemia (20.9%), and persistent ketonuria (20.5%).
Dicks and Naidoo (39), 2022	South Africa	“COVID-19 and diabetic ketoacidosis: A case series at an urban district hospital in South Africa.”	Patients with DKA and confirmed COVID-19 (n = 10)	Retrospective	− All patients fulfilled diagnostic criteria for DKA and presented with severe metabolic derangement. − Many had concurrent COVID-19 symptoms, with profound metabolic acidosis at admission (mean pH 7.02; range 6.87–7.29). Half had known T2DM, while the remainder were newly diagnosed at presentation. − COVID-19, chronic poor glycemic control (HbA1c 9.7–13.8%), and treatment inertia—particularly reliance on metformin monotherapy in patients with known T2DM—were key precipitants of DKA. Hypoxia at admission strongly predicted mortality. − The case fatality rate was 30% (3/10), with deaths occurring mainly among patients with severe metabolic acidosis and hypoxia.
Eskeziya et al. (47), 2020	Ethiopia	“Prevalence of diabetic keto acidosis and associated factors among newly diagnosed patients with type one diabetic mellitus at Dilla University Referral Hospital, September 9th/2017 – May 30th/2019: South Ethiopia; Cross-sectional study.”	Newly diagnosed T1DM adults (n = 421)	Cross-sectional	− Among newly diagnosed T1DM patients, DKA prevalence was 38% (160/421). − Common presentations included polyuria/polydipsia (62.9%), dehydration with dry mouth (30.2%), and altered mental status (27.8%), with vomiting and fast breathing indicating advanced metabolic decompensation at diagnosis. − Increased odds of DKA were associated with young adulthood (18–25 years; AOR 3.1) and preceding infection (AOR 46), particularly urinary tract infection, cough, and headache. − Family history of diabetes and prior knowledge of diabetes symptoms were protective.
Gebre and Assefa (48), 2019	Ethiopia	“Magnitude and associated factors of diabetic complications among diabetic patients attending Gurage zone hospitals, South West Ethiopia.”	DM patients (n = 338)	Cross-sectional	− Diabetes-related complications affected 61% (206/338) of patients. Acute hyperglycemic emergencies accounted for 26.9%, including DKA (14.2%), HHNS (6.2%), and hypoglycemia (6.5%). − Complications occurred across both diabetes types, with a balanced distribution between type 1 (47.3%) and T2DM (52.7%). − Increased odds of complications were associated with poor glycemic control (AOR 1.88), overweight/obesity (BMI ≥25; AOR 4.42), and longer diabetes duration (6–10 years: AOR 1.79; >10 years: AOR 4.68). Being divorced was associated with lower odds (AOR 0.25). − Chronic complications predominated (60.7%), most commonly nephropathy (20.4%), diabetic foot ulcers (16.9%), neuropathy (14.8%), and retinopathy/visual impairment (14.5%). − Marked metabolic dysregulation, including elevated fasting glucose (89.6%), random glucose (81.1%), and LDL cholesterol (49.7%), despite moderate lifestyle adherence.
Gebre et al. (49), 2023	Ethiopia	“Treatment outcome and its predictors among diabetic patients attending selected hospitals of Southern Ethiopia.”	Adult DM patients (n = 422)	Cross-sectional	− Diabetic emergencies included: DKA: 195/422 (46.2%); HHNKS: 21/422 (5.0%); hypoglycemia: 7/422 (1.7%); any acute emergency (DKA + HHNKS + hypoglycemia): 223/422 (52.8%). Most participants had T2DM (272/422, 64.5%), followed by T1DM (143/422, 33.9%) and other types (7/422, 1.7%). − Grade 1–8 education (COR 1.72), prior diabetes-related admission (COR 1.69), hyperglycemia versus DKA complications (COR 0.47), daily blood glucose monitoring (COR 0.26), and elevated fasting glucose (COR 0.14). − 277 of 422 patients (65.6%) had a good treatment outcome, defined as an average fasting blood glucose of 80–130 mg/dl, while 145 (34.4%) had a poor treatment outcome with fasting blood glucose above 130 mg/dl. − DKA was associated with better outcomes compared with hyperglycemia (COR 0.465; 95% CI 0.259–0.833)
Gebremedhin et al. (40), 2021	Ethiopia	“Hyperglycemic crisis characteristics and outcome of care in adult patients without and with a history of diabetes in Tigray, Ethiopia: Comparative study.”	DM patients (n = 589)	Retrospective	− Hyperglycemic crises were frequent and severe, with DKA in 48.7%, HHS in 31.8%, uncomplicated hyperglycemia in 22.8%, and severe hypoglycemia in 22.3%. − DKA predominated among patients without prior diabetes (84.2%), whereas HHS was more common in those with known diabetes (31.8%). − Presentations commonly included vomiting (69.4%, abdominal pain (24%), polyuria (75.5%), polydipsia (81.1%), weight loss (79.6%), fruity breath (84.2%), and altered consciousness (26.9%). − Higher odds were associated with rural residence (AOR 3.1), prior stroke (AOR 2.7), T2DM (AOR 2.3), HHS presentation (AOR 2.4), and known diabetes (AOR 2.0). Infection (40.1%) and treatment non- adherence (26.4%) were common precipitants, while polydipsia was protective (AOR 0.47). − Patients without prior diabetes had lower mortality (8.7%) than those with known diabetes (15.5%). − Mortality was substantially higher in HHS compared with DKA (27–29% vs 4.8–10.1%). − Mortality was higher among rural patients and those with cardiovascular disease, chronic kidney disease, prior stroke, or low urine ketones.
Getie et al. (24), 2021	Ethiopia	“Determinants of diabetes ketoacidosis among diabetes mellitus patients at North Wollo and Waghimra zone public hospitals, Amhara region, Northern Ethiopia.”	Adult DM patients (n = 408 [102 DKA cases, 306 controls]).	Unmatched case–control study	− All cases presented with DKA, fulfilling diagnostic criteria (plasma glucose ≥250 mg/dL, ketonemia/ketonuria, and reduced serum bicarbonate), often with severe metabolic acidosis and concurrent infections or comorbidities. − T1DM accounted for 43.1% of DKA cases; the study focused on determinants rather than population prevalence. − Independent risk factors included irregular clinic follow-up (AOR 4.19), lack of prior diabetes education (AOR 2.87), alcohol consumption (AOR 2.99), medication discontinuation (AOR 4.31), comorbidities (AOR 2.57), and T1DM (AOR 2.01).
Iloh and Amadi (11), 2018	Nigeria	“Epidemiology of diabetic emergencies in the adult emergency department of a tertiary hospital in South-Eastern Nigeria.”	Adult DM patients (n = 156)	Retrospective	− Hyperglycemic crises were common, with HHS accounting for 50.6%, DKA for 41.6%, and hypoglycemic crises for 7.8%. − Classic hyperglycemic symptoms were frequent, with 3P symptoms reported by 50.3% and 2P combinations by 29.2%. DKA occurred more often in T1DM (28.2%) than in T2DM (11.8%). − Hypertension was the most prevalent comorbidity (58.3%), underscoring the role of cardiometabolic comorbidity in precipitating emergencies. − Crises predominantly affected young and middle-aged adults (18–59 years). − Presentations occurred mainly at night (56.4%) and during the dry season (52.6%).
Iradukunda et al. (41), 2021	Rwanda	“Diabetic complications and associated factors: a 5-year facility-based retrospective study at a tertiary hospital in Rwanda.”	DM patients (n = 246)	Retrospective	− Diabetes-related complications were frequent, including hypertension (50.4%), acute hyperglycemic states (24%), nephropathy (23.6%), and stroke (15.4%). − Nearly one-quarter experienced an acute hyperglycemic emergency, predominantly among T2DM (84.5%), with T1DM accounting for 15.5%. − Alcohol consumption (OR 8.0), older age (≥45 years; OR 6.2), and T1DM (OR 14.1) were strong predictors. Longer diabetes duration was also associated (p = 0.001), and poor glycemic control was present in 95.2% of those with complications. − Acute emergencies co-occurred with a high burden of chronic comorbidities, notably nephropathy and cerebrovascular disease. − The frequent coexistence of hypertension and diabetes compounds cardiovascular risk and clinical complexity.
Kefale et al. (54), 2016	Ethiopia	“Hospitalization pattern and treatment outcome among diabetic patients admitted to a teaching hospital in Ethiopia: A prospective observational study.”	DM patients (n = 89)	Prospective	− Nearly 48.3% (43/89) of hospital admissions were diabetes related. DKA was the leading cause (33.7%), followed by infections (19.1%)—notably diabetic foot ulcers (15.7%) and bacterial meningitis (4.5%)—and cardiovascular diseases (18.0%), mainly congestive heart failure (14.6%) and stroke (3.4%). HHS accounted for 4.5% of admissions. − Most patients had T2DM (74.2%), and 37.1% were newly diagnosed at admission. − Admission due to DKA was strongly associated with diabetes-related hospitalization (COR 4.0). Comorbidities were common (66.3%), particularly hypertension (34.8%) and infections (24.7%). Admissions complicated by bacterial meningitis were linked to poorer outcomes (COR 0.9). − Patients admitted with diabetic complications were more likely to have favorable outcomes (AOR 5.7). − Mortality was 11.2%, and 76.4% were discharged with clinical improvement. − Median hospital stay was 9 days (range 1–88), with markedly elevated admission glucose (347.8 ± 158.8 mg/dL). − Chronic complications occurred in 24.7%, mainly diabetic foot ulcers (11.2%), peripheral neuropathy (7.9%), nephropathy (4.5%), and retinopathy (3.4%).
Kidanie et al. (53), 2020	Ethiopia	“Determinants of diabetic complication among adult diabetic patients in Debre Markos Referral Hospital, Northwest Ethiopia, 2018: Unmatched case control study.”	Adult DM patients (n = 204 [68 cases with at least one complication, 136 controls])	Unmatched case–control study	− Patients experienced both acute (hypoglycemia, hyperglycemia/DKA, HHNS) and chronic complications (nephropathy, diabetic foot ulcers). − Although prevalence was not estimated, 61.8% of participants had poor glycemic control, and T1DM accounted for 57.8% of the cohort. − Higher odds of complications were associated with older age (38–47 years: AOR 5.60; ≥47 years: AOR 4.81), lower monthly income (1000–1499 ETB; AOR 3.10), poor treatment adherence (AOR 5.15), and T1DM (AOR 4.73). Good fasting blood glucose control was strongly protective. − The coexistence of acute metabolic events and chronic end-organ complications indicates a substantial clinical burden driven by sustained hyperglycemia and suboptimal disease management.
Kidie et al. (31), 2021	Ethiopia	“Frequency of diabetic ketoacidosis and its determinants among pediatric diabetes mellitus patients in Northwest Ethiopia.”	Pediatric DM patients (n = 389)	Retrospective	− Recurrent DKA was common and life-threatening among children with diabetes, with a mean of 1.01 DKA episodes per child during follow-up. − Higher DKA frequency was associated with intercurrent infections (AIRR 1.41), underlying heart disease (AIRR 4.10), treatment discontinuation (AIRR 2.91), and low serum sodium (AIRR 1.88). Higher baseline insulin dosing was protective (AIRR 0.96 per unit increase). − Recurrent DKA episodes contributed to significant morbidity and clinical instability, confirming DKA as a major driver of adverse outcomes in pediatric diabetes.
Lontchi‐Yimagou et al. (50), 2017	Cameroon	“Ketosis-prone atypical diabetes in Cameroonian people with hyperglycemic crisis: frequency, clinical and metabolic phenotypes.”	Adults with non-autoimmune diabetes (n = 173 participants: 124 type 2 DM, 49 ketosis-prone diabetes).	Cross-sectional	− Hyperglycemic crises occurred with and without ketosis. Ketosis-prone diabetes (KPD) accounted for 28.3% (49/173) of crises, with most presenting as acute ketotic episodes. − Significant weight loss >5% at diagnosis was common (73.5%). The cohort had a high cardiometabolic burden, with a mean BMI of 27.7 kg/m² and overweight/obesity in 55.8%. − KPD was characterized by new-onset diabetes without typical precipitants (infection, stress, corticosteroids) but with marked ketosis requiring insulin. During ketotic episodes, patients showed reduced insulin secretion and higher triglyceride levels, indicating transient severe insulin deficiency.
Lotter et al. (7), 2021	South Africa	“The burden of diabetic emergencies on the resuscitation area of the district-level public hospital in Cape Town.”	DM patients (n = 197)	Retrospective	− Diabetic emergencies affected 8.1% (197/2,424) of patients. DKA was the most common presentation (48.7%), followed by uncomplicated hyperglycemia (22.8%), severe hypoglycemia (22.3%), and HHS (6%). − Infection was the leading precipitating factor (40.1%), with an identifiable trigger established in 88% of cases, highlighting the importance of preventable causes. − Acute kidney injury occurred in 40.6% of cases. − Median resuscitation-area stay was 13 hours (IQR 7.2–24). − Recurrent emergencies were frequent (51.3% within six months), and overall mortality was 5% (n = 10).
Ndebele and Naidoo (42), 2018	South Africa	“Management of diabetic ketoacidosis at a rural regional hospital in KwaZulu-Natal.”	DKA patients (n = 105)	Retrospective	− DKA was the predominant emergency. Most cases occurred in T1DM (60.9%), though T2DM accounted for 39.1%. Presentations were mainly moderate (56.2%) or severe (24.8%), with 19.1% mild. Common features included lethargy with vomiting (31.4%), isolated lethargy (11.4%), reduced consciousness (10.5%), and septic limb manifestations (5.7%). − Infection and poor treatment adherence were frequent precipitants, with a trigger identified in 64.8% of cases. Infection was more common in T2DM (OR 11.13, 95% CI 3.86–33.49). Hypertension was the most prevalent comorbidity (24.8%), and glycemic control was poor (mean HbA1c 13.8%). − Mean resuscitation-unit stay was 1.51 ± 0.8 days, with total hospitalization of 8.9 ± 7.5 days. − Mortality was 17.1% (18/105), predominantly among T2DM patients, and deaths occurred in older patients (47.2 ± 12.8 vs 36.6 ± 16.8 years; p = 0.01).
Negera et al. (51), 2020	Ethiopia	“Acute complications of diabetes and its predictors among adult diabetic patients at Jimma Medical Center, Southwest Ethiopia.”	Adult DM patients (n = 348)	Cross-sectional	− Acute hyperglycemic complications occurred in 26.4% (92/348) of patients. DKA was predominant (73.9%), followed by HHS (22.8%) and hypoglycemia (3.3%). Most events involved T2DM (80.7%), with T1DM accounting for 19.3%. − Increased risk was associated with comorbidities (AOR 5.6), T1DM (AOR 9.3), uncontrolled blood glucose (AOR 1.91), and limited access to health facilities (AOR 1.96), indicating both clinical and health-system determinants.
Oromo (43), 2025	Ethiopia	“Pediatric Diabetic Ketoacidosis (PDKA) among newly diagnosed diabetic patients at Dilla University Hospital, Dilla, Ethiopia: Prevalence and predictors.”	Newly diagnosed T1DM children (n = 61)	Retrospective	− DKA was a frequent initial presentation among children with newly diagnosed T1DM, occurring in 60.7% (37/61). − Presentations indicated advanced metabolic decompensation, including vomiting with dehydration (37.8%), fast breathing (29.7%), abdominal pain (27.2%), polyuria/polydipsia with weight loss (26.2%), and loss of consciousness (10.8%). − Preceding infection markedly increased DKA risk (AOR 11.69), commonly pneumonia, diarrheal illness, UTI, and tonsillopharyngitis. Protective factors included parental knowledge of diabetes symptoms (AOR 0.07), family history of diabetes (AOR 0.13), and higher family income.
Thomas et al. (30), 2019	South Africa	“Audit of diabetic ketoacidosis management at a tertiary hospital in Johannesburg, South Africa.”	DKA patients (n = 69)	Prospective	− Approximately 26.7 DKA admissions per month; nearly one−third present with DKA as the first manifestation of diabetes, and over one−third report previous DKA episodes, indicating substantial recurrent emergency burden. − Non-adherence was the commonest precipitant (47.14%), followed by sepsis (15.71%) and other/unknown causes (37.14%); severity of DKA (by pH category) was the only independent predictor of longer time to resolution (OR 4.89, 95% CI 1.04–22.84). − All 69 DKA episodes resolved with hourly IV bolus insulin − Early survival 100%, and complications occurred in 12.68% (mainly procedures for septic foci, aspiration pneumonia, GI bleeding) − The mean time to resolution was 21 (13.5-29) hours − , and severe DKA was associated with longer resolution times.
YimamAhmed et al. (52), 2020	Ethiopia	“Glycemic control, diabetes complications and their determinants among ambulatory diabetes mellitus patients in Southwest Ethiopia: A prospective cross-sectional study.”	Ambulatory DM patients (n = 100)	Cross-sectional	− Chronic diabetes-related morbidity predominated, with 59% developing at least one chronic complication. Peripheral neuropathy was most common (69.5%), followed by impotence (8.5%), retinopathy (8.5%), and diabetic foot ulcers (6.8%). − Poor glycemic control was widespread, with 71% having uncontrolled fasting blood glucose, indicating a high risk of hyperglycemic emergencies. − Mean fasting glucose was 158.8 ± 47.6 mg/dL, and 40% had comorbidities, mainly hypertension (82.5%). − Low medication adherence was a major determinant (AOR 11.78). Inappropriate dosing during clinic visits was strongly associated with adverse outcomes (first visit: AOR 7.70; second visit: AOR 8.09; third visit: AOR 4.34). Most participants had T2DM (76%).

### 3.2 Type and prevalence of acute emergencies

Across the included African studies, diabetic emergencies were common, with DKA emerging as the predominant hyperglycemic emergency, followed by HHS and hypoglycemia (**Table 2**). Seventeen studies documented the prevalence/rate of DKA among diabetic patients (7, 11, 25, 31, 36–38, 40, 43–45, 47–51, 54). The reported proportions of DKA varied widely, ranging from 2.0% among adults with T2DM in Ghana (36) to 92.6% among patients admitted for hyperglycemic emergencies in Ethiopia (38). Other studies also indicated significant proportions of DKA, including 40.0% among diabetic admissions in Hawassa (37) and 46.2% among diabetic patients in Southern Ethiopia (49). Two studies reported a DKA prevalence of 48.7% among those experiencing hyperglycemic crises, respectively in Tigray and Cape Town (7, 40), and in Nigeria, DKA constituted 41.6% of diabetic emergencies (11).

**Table 2:** Type and prevalence of diabetic emergencies.

Type of diabetic emergency	Number of studies	Prevalence/rates (%)	Authors
Diabetic Ketoacidosis (DKA)	17	2.0 to 92.6%	(7, 11, 25, 31, 36–38, 40, 43–45, 47–51, 54).
Hyperosmolar Hyperglycemic State (HHS)	11	4.5 to 50.6%	(7, 11, 25, 37, 38, 40, 44, 48, 49, 51, 54)
Hypoglycemia	9	0.8 to 28.3%	(7, 11, 36, 37, 40, 44, 48, 49, 51)

DKA was notably prevalent among newly diagnosed patients as reported in four studies (37, 38, 43, 47), affecting 38.0% of adults with T1DM in a study by Eskeziya et al. (47) and 53.9% of newly diagnosed patients in Bedaso et al. (37), while Desse et al. (38) reported 23.6% among patients with newly diagnosed diabetes. A study by Oromo (43) found that among children with newly diagnosed T1DM, DKA occurred in 60.7% of cases. Two studies focused on pediatric populations, both from Ethiopia, and both concentrated on DKA rather than the full spectrum of acute events (31, 43). In a larger pediatric follow-up cohort (31), 94.6% had DKA, with 48.3% experiencing at least one DKA episode during follow-up, whereas a study by Oromo (43) reported that 37 of 61 newly diagnosed children with T1DM had DKA. The mean frequency of DKA was 1.01 episodes per child, highlighting a significantly high acute DKA burden in children (31). Furthermore, four studies focused on hospital-based cohorts (24, 30, 39, 42). One study by Lontchi‐Yimagou et al. (50) reported that Ketosis-prone diabetes (KPD) accounted for 28.3% of crises, with most presenting as acute ketotic episodes. Abate et al. (25) reported a DKA incidence rate of 12.5 per 100 person-years, noting that it was significantly more common among individuals with T1DM (35.6%) compared to those with T2DM (6.3%).

The HHS prevalence was reported in 11 studies (7, 11, 25, 37, 38, 40, 44, 48, 49, 51, 54). The proportions of HHS varied, ranging from 4.5% of diabetes-related admissions in Ethiopia (54) to 50.6% of diabetic emergencies in Nigeria (11). In other studies, HHS comprised 7.4% of hyperglycemic emergencies in Southwest Ethiopia (38), 7.9% of acute complications in Northeast Ethiopia (44), and 9.0% of acute complications in Hawassa (37). Additionally, it accounted for 6.2% of acute hyperglycemic emergencies in Gurage zone hospitals (48), 5.0% of diabetic emergencies in Southern Ethiopia (49), 6.0% of diabetic emergencies in Cape Town (7), and 22.8% of acute hyperglycemic complications at Jimma Medical Center (51). In the Tigray cohort, HHS represented 31.8% of hyperglycemic crises and was more prevalent among patients with known diabetes (40). Abate et al. (25) reported that the incidence of HHS was 2.1 per 100 person-years, mostly occurring in patients with T2DM.

Hypoglycemia was documented in nine studies (7, 11, 36, 37, 40, 44, 48, 49, 51). The reported proportions varied, ranging from 0.8% in a large cohort of T2DM patients in Ghana (36) to 28.3% of acute complications in Ethiopia (44). Other studies indicated that hypoglycemia accounted for 14.0% of acute complications in the emergency unit of Hawassa University Comprehensive Specialized Hospital (37), 6.5% of acute hyperglycemic emergencies in hospitals in the Gurage zone (48), 1.7% of diabetic emergencies in Southern Ethiopia (49), 7.8% of diabetic emergencies in Nigeria (11), and 3.3% of acute hyperglycemic complications at Jimma Medical Center (51). Severe hypoglycemia represented 22.3% of diabetic emergencies in both the Tigray and Cape Town hospital cohorts (7, 40).

### 3.3 Predisposing factors to diabetic emergencies

Predisposing factors to diabetic emergencies and diabetic complications were identified, including type of diabetes (T1DM), poor glycemic control, infections, non-adherence to treatment, comorbidities, age, diabetes duration, socioeconomic status, access to care, knowledge and education, biochemical and clinical markers, and behavioral factors (**Table 3**). Nine studies reported that T1DM was associated with increased odds of hyperglycemic emergencies, particularly DKA, compared with T2DM (25, 40–45, 51, 53). For example, Abate et al. (25) reported a 2.75-fold increased risk of hyperglycemic emergencies in T1DM, while Birhanu et al. (45) found that patients with T1DM had a significantly higher risk of developing DKA compared with those with T2DM (AOR = 3.106; 95% CI: 1.150–7.273). Birhanu et al. (45) also reported that 71.4% of DKA patients had T1DM, while 28.6% had T2DM. Similarly, Negera et al. (51) found that 73.9% of acute complications in T1DM patients were DKA.

**Table 3:** Predisposing factors to diabetic emergencies.

Predisposing factors	Number of studies	Description of predisposing factors to diabetic emergencies	Authors
T1DM	9	− T1DM is consistently associated with higher odds of hyperglycemic emergencies, especially DKA, compared with T2DM. − Risk was elevated both at diagnosis and in established disease.	(25, 40–45, 51, 53).
Poor glycemic control	8	− High HbA1c or uncontrolled fasting glucose strongly predicted both incident and prevalent acute complications. − The majority of acute complications had poor control.	(25, 41, 46, 48, 49, 51–53).
Infections	11	− Urinary tract infection (UTI) − Pneumonia and other respiratory infections − Sepsis − Diarrhea − Infections (not specified)	(7, 25, 30, 31, 38, 40, 42, 43, 45–47)
Non-adherence and treatment gaps	10	− Missed appointments − Medication non-adherence − Treatment discontinuation − Infrequent follow-up	(24, 25, 30, 31, 38, 40, 46, 51–53)
Comorbid conditions	9	− Hypertension, cardiovascular disease, renal impairment, heart disease, and other comorbidities substantially increased both the risk and severity of hyperglycemic emergencies.	(25, 31, 37, 38, 40, 46, 48, 51, 54)
Age	6	− Younger adults (around 20–29 years) were more likely to develop DKA − Older age groups (≥38–47 years and above) had higher odds of overall diabetic complications, including acute events.	(36, 37, 41, 44, 45, 53)
Duration of diabetes	5	− A longer duration (≥5–10 years) was associated with a greater overall complication burden.	(25, 36, 41, 48, 53)
Socioeconomic and access factors	7	− Unemployment − Low income − Rural residence − Lack of health insurance, − Long distance to facilities	(25, 36, 40, 43, 45, 51, 53)
Knowledge and education	3	− Lack of diabetes education − Low parental knowledge about diabetes symptoms	(24, 43, 47)
Behavioral factors	5	− Tobacco use − Alcohol consumption	(24, 41, 45, 46, 49)
Biochemical and clinical markers	4	− Low serum sodium − Elevated creatinine − Hypoxia − Sepsis − Overweight/obesity	(31, 38, 39, 48)

In six studies, age was linked with diabetic emergencies (36, 37, 41, 44, 45, 53). Results from Iradukunda et al. (41) and Kidanie et al. (53) revealed that younger adults, especially those aged 20–29 years, were more susceptible to DKA, whereas older age groups (38–47 years and older) had heightened odds of overall diabetic complications. In pediatrics, two studies reported the relationship between T1DM and DKA (31, 43). A study by Kidie et al. (31) reported that 94.6% of children with T1DM presented with DKA at onset, with 48.3% experiencing recurrences, while Oromo (43) found that 60.7% of admitted T1DM children were DKA cases.

Five studies established a relationship between diabetes duration and diabetic complications (25, 36, 41, 48, 53), especially 5–10 years or more. A study by Annani-Akollor et al. (36) reported odds ratios of 1.55 and 2.76 for complications in longer T2DM durations. Eight studies reported that high HbA1c levels and uncontrolled fasting blood glucose were correlated with both incident and prevalent acute complications (25, 41, 46, 48, 49, 51–53). A study by Cedrick et al. (46) reported that 78% of participants had HbA1c levels ≥7.0%, with poor glycemic control linked to missed appointments, comorbidities, and infections. Furthermore, Negera et al. (51) found uncontrolled blood glucose associated with acute complications (AOR 1.91).

Across 11 studies, infections such as urinary tract infections, pneumonia, sepsis, diarrheal diseases, and respiratory infections were major triggers for hyperglycemic emergencies (7, 25, 30, 31, 38, 40, 42, 43, 45–47). A study by Desse et al. (38) found infections in 59% of cases, while Lotter et al. (7) reported 40.1% of cases had infections, with 88% of precipitating factors identifiable. Furthermore, a study by Birhanu et al. (45) found that infection increased the risk of DKA (AOR = 2.819; 95% CI 1.138–8.428). Similarly, a pediatric study by Kidie et al. (31) found that infections were associated with increased DKA frequency (AIRR 1.41).

Comorbidities associated with diabetic emergencies, including hypertension, cardiovascular disease, and renal impairment, were discussed in nine studies (25, 31, 37, 38, 40, 46, 48, 51, 54). For instance, Desse et al. (38) reported that comorbidities increased mortality odds by over 15-fold (AOR 15.26), while elevated creatinine levels were associated with mortality (AOR 5.86). A study by Kidie et al. (31) found that patients with heart disease as a comorbidity were 4.1 times more likely to experience frequent episodes of DKA than those without heart disease (AIRR 4.1, 95% CI 1.17–14.68).

Ten studies reported non-adherence and treatment gaps as predisposing factors to diabetic emergencies (24, 25, 30, 31, 38, 40, 46, 51–53). Thomas et al. (30) found that 47.14% of cases involved treatment non-adherence, compared with 26.4% reported by Gebremedhin et al. (40). Furthermore, Getie et al. (24) reported that irregular follow-up (AOR 4.19) and treatment discontinuation (AOR 4.31) correlated with DKA. Similarly, Abate et al. (25) found that infrequent follow-up increased the risk of hyperglycemic emergency (AHR 1.79).

In 7 studies, socioeconomic and access-related factors were associated with increased risk of diabetic emergencies (25, 36, 40, 43, 45, 51, 53). A study by Kidanie et al. (53) found that an income of 1,000–1,499 ETB was associated with over threefold increased complication odds (AOR 3.10). Similarly, Annani-Akollor et al. (36) reported that 59.0% of 1,600 adults with T2DM had complications, with employed individuals having lower complication odds than unemployed individuals. A study by Negera et al. (51) noted that poor access to healthcare facilities was linked to acute complications (AOR 1.96), while Gebremedhin et al. (40) found rural residence associated with mortality.

Three studies reported that lack of diabetes education and low parental knowledge were associated with increased DKA risk at diagnosis, while awareness of diabetic symptoms and family history of diabetes were protective factors (24, 43, 47). A study by Eskeziya et al. (47) found symptom awareness reduced DKA odds (AOR 0.825), and a family history of diabetes was linked to lower DKA odds at onset (AOR 0.16). Similarly, Oromo (43) reported that parental awareness reduced pediatric DKA by 93% (AOR 0.07), and family history reduced risk by 87% (AOR 0.129). However, a study by Getie et al. (24) found diabetes mellitus patients who had not received diabetes education were 2.87 times more likely to develop diabetic ketoacidosis than those who had received such education [AOR 2.87, 95% CI 1.44–5.72].

Five studies identified that behavioral factors, such as tobacco use and alcohol consumption, were associated with poor glycemic control and acute hyperglycemic states (24, 41, 45, 46, 49). For instance, Iradukunda et al. (41) reported that alcohol use increased complication odds eightfold (OR 8.0), while Cedrick et al. (46) found tobacco use linked to poor glycemic control (AOR 2.01). Similarly, Getie et al. (24) found that the likelihood of developing DKA was 2.99 times higher in diabetes mellitus patients who consumed alcohol compared with those who did not (AOR 2.99). Birhanu et al. (45) noted that of 82 DM patients who used alcohol, 14.6% had DKA. However, a study conducted by Gebre et al. (49) found no significant association between alcohol use or smoking and poor treatment outcomes. In their study, Gebre et al. (49) found that alcohol users accounted for 4.8% of poor outcomes (OR: 0.815; 95% CI: 0.309–2.150), while smokers made up 3.4% (OR: 0.307; 95% CI: 0.072–1.301).

Four studies reported that biochemical and clinical markers, such as low serum sodium, elevated creatinine, sepsis, hypoxia at admission, and overweight or obesity, were indicators of severe diabetic emergencies and adverse in-hospital outcomes (31, 38, 39, 48). A study by Desse et al. (38) reported that elevated creatinine was associated with mortality, while Kidie et al. (31) found that patients with low sodium levels had an 88% higher incidence rate of DKA compared with those with normal sodium (AIRR 1.88, 95% CI 1.22–2.89). Gebre and Assefa (48) found that having a body mass index greater than 25 was associated with higher odds of developing diabetic complications [AOR 4.42, 95% CI 1.32–14.86]. A study by Dicks and Naidoo (39) found that among patients with DKA and COVID-19, three patients died, two of whom were hypoxic on admission.

### 3.4 Clinical outcomes of diabetic emergencies

Across the included African studies, diabetic emergencies were associated with substantial in-hospital mortality, prolonged admissions, acute and chronic complications, and high recurrence, while most survivors were discharged improved but often with chronic diabetic organ damage and elevated cardiovascular risk (**Table 4**). Six studies showed that the in-hospital mortality ranged from approximately 5% to 17.1% overall, with DKA-specific case fatality rates of 9.8% to 30% (7, 38–40, 42, 54). Two studies from Ethiopia (38, 54) reported that mortality rates varied from 9.8% (38) to 11.2% (54) in tertiary teaching hospitals. In South Africa, three studies reported a varying degree of mortality (7, 39, 42). A study by Ndebele and Naidoo (42) reported a DKA mortality rate of 17.1% with an average hospitalization of 8.9 days, whereas mixed emergency centers noted a 5.1% mortality rate (7). Additionally, COVID-19–associated DKA cases in South Africa showed a 30% mortality rate, particularly among patients with severe acidosis and hypoxia (39).

**Table 4:** Clinical outcomes of diabetic emergencies.

Clinical outcome	Number of studies	Key points from included studies	Authors
In-hospital mortality	6	− Mortality ranged from 5–17.1% overall − DKA-specific case fatality rates were between 9.8% and 30% − Higher among T2DM and older adults (>47 years) − Mortality was higher in rural residents compared with urban patients − Risk of death was increased in those with cardiovascular, kidney, or cerebrovascular comorbidities	(7, 38–40, 42, 54).
Length of hospital stay	4	− Median length of hospital stay ranged from 6 to 9 days − Average for resuscitation unit stay was 1.5 days	(7, 38, 42, 54)
Time to metabolic resolution	2	− Average time to resolution was 21 (13.5–29) hours − DKA: 64.4 ± 76.3 hours − HHS: 29.0 ± 20.6 hours	(30, 38)
Recurrence	3	− Recurrent hyperglycemia (54%), hypoglycemia (20.9%) − Persistent ketonuria (20.5%) during DKA management. − 51.3% returned with a diabetic emergency within 6 months − Recurrent DKA was common in children (mean 1.01 episodes).	(7, 31, 38)
Favorable discharge/improvement	2	− 76.4% of patients were discharged in an improved condition − Positive treatment outcome in 65.6% − Complications at admission predicted a better outcome (AOR 5.7).	(49, 54)
Coexisting complications	11	− Acute kidney injury − Foot ulcers − Retinopathy − Nephropathy − Hypertension − Neuropathy − Stroke	(7, 36–38, 41, 44, 45, 48, 52–54)

Two studies explicitly reported favorable discharge or clinical improvement following diabetic emergencies (49, 54). A study by Kefale et al. (54) reported that 76.4% of patients were discharged in an improved condition, while Gebre et al. (49) found that 65.6% achieved a positive treatment outcome.

Four studies reported that diabetic emergencies were associated with longer stays in hospital and resuscitation units (7, 38, 42, 54). A study by Desse et al. (38) reported that the median hospital stay was 6 days, Kefale et al. (54) reported a median hospital stay of 9 days (range 1–88), while Ndebele and Naidoo (42) reported a total hospitalization of 8.9 ± 7.5 days. In the resuscitation unit, a study by Lotter et al. (7) found that the median stay in the resuscitation area was 13 hours (IQR 7.2–24), while Ndebele and Naidoo (42) reported a mean resuscitation-unit stay of 1.51 ± 0.8 days. Two studies quantified the time to metabolic resolution of diabetic emergencies (30, 38). A study by Thomas et al. (30) found that the mean time to resolution was 21 (13.5–29) hours, and severe DKA was associated with longer resolution times. Desse et al. (38) reported that mean durations of treatment until resolution were 64.38 ± 76.34 hours for DKA and 29.00 ± 20.58 hours for HHS.

Three studies described instances or patterns of diabetic emergencies recurring (7, 31, 38). Notably, Desse et al. (38) documented recurrent dysglycemia during admission, specifically recurrent hyperglycemia in 54% of patients, hypoglycemia in approximately 20.9%, and persistent ketonuria in 20.5% despite active management (38). Furthermore, a study by Lotter et al. (7) reported that 51.3% of patients returned with a diabetic emergency within six months, while Kidie et al. (31) found that among children, 48.3% had at least one additional DKA episode, with some experiencing up to six episodes over two years.

Eleven studies described coexisting complications among patients with diabetic emergencies (7, 36–38, 41, 44, 45, 48, 52–54). A study by Abejew et al. (44) found that chronic complications were more frequent than acute (58.8% vs 41.2%), with hypertension (43.3%), visual disturbances (28.9%), neuropathy (14.4%), foot ulcers (4.4%), and nephropathy (2.2%). In a study by Lotter et al. (7), acute kidney injury was observed in up to 40.6% of admissions, while Gebre and Assefa (48) reported that chronic complications accounted for 60.7%. Similarly, Bedaso et al. (37) reported a high rate of chronic complications, including diabetic foot ulcers (60%), retinopathy (27%), and nephropathy (13%). YimamAhmed et al. (52) found that 59% of chronic diabetes patients experienced at least one chronic complication, most commonly peripheral neuropathy (69.5%).

## 4 Discussion

This scoping review revealed a high burden of diabetic emergencies in Africa, predominantly characterized by hyperglycemic crises (25, 37, 41, 44, 48, 49, 51). DKA was the most frequent emergency (2.0%–92.6%), particularly among young and T1DM patients (7, 25, 36, 38, 40, 51). In contrast, HHS, while less common than DKA, was more prevalent among older adults and patients with T2DM (4.5%–50.6%) (7, 11, 25, 37, 38, 40, 44, 48, 49, 51, 54). Hypoglycemia was less frequently reported (0.8%–28.3%) compared to hyperglycemic crises (7, 11, 36, 37, 40, 44, 48, 49, 51). More importantly, the findings suggest that DKA, HHS, and hypoglycemia often arise within a broader risk continuum shaped by factors such as poor glycemic control, recurrent infections, inconsistent follow-up, and limited access to structured diabetes education (7, 24, 25, 40, 45, 53). Uncontrolled blood glucose levels were a central factor in many diabetic emergencies, highlighting that these emergencies often stem from prolonged suboptimal diabetes management rather than sudden deterioration (25, 41, 46, 48, 49, 51–53). Furthermore, this review showed that missed appointments and poor medication adherence contributed to acute diabetic crises by disrupting routine care (24, 30, 38, 46). Infection and comorbidity significantly affected the onset and severity of diabetic emergencies, with acute metabolic crises often occurring amid broader medical instability (7, 38, 45, 46). Clinical outcomes of diabetic emergencies were associated with significant short-term morbidity and mortality. High in-hospital mortality rates, prolonged hospital stays, and recurrent hyperglycemia indicate a burden on inpatient services, often reflecting underlying deficiencies in diabetes management in the African context (7, 38–40, 42, 54).

The patterns of diabetic emergencies in Africa are comparable to global trends. In developed countries, DKA represents a significant proportion of acute diabetes-related admissions (4, 5, 55). In Europe, the US, and Israel, DKA incidence was between 0 and 56 cases for every 1,000 adults with T1DM each year (56). A study conducted in China among 158 patients with T2DM by Wu et al. (55) reported a prevalence of 41.1% for DKA, 46.8% for HHS, and 12.0% for mixed DKA-HHS. A systematic review by Haile and Fenta (4) reported that, among hospitalized diabetic patients in developed countries such as Europe, Australia, New Zealand, and the US, the mean prevalence of DKA was 29.9%, ranging from 19.5% in Sweden to 43.8% in Luxembourg. Dhatariya et al. (56) reported that in developing countries, DKA was between 3.8% and 73.4% of people living with diabetes. Furthermore, a population-based study in Switzerland by Ebrahimi et al. (8) found that DKA incidence increased from 7.22 per 100,000 person-years in 2010 to 9.49 per 100,000 person-years in 2018, particularly among younger individuals and those with T1DM. In contrast, among individuals with T2DM, DKA incidence remained below 1 event per 100,000 person-years until age 37, after which it increased progressively, reaching 12.86 events per 100,000 person-years in females aged 87 and 13.11 per 100,000 person-years in males aged 82 (8). Regarding hypoglycemic crises, a study conducted in the US by Galindo et al. (57) found that younger patients aged 18–44 years had the highest rate of hypoglycemic crises, with 120.07 events per 1,000 person-years (95% CI: 114.84–125.30), compared with 42.07 events per 1,000 person-years among patients aged 75 years and older. In Japan, Ikeda et al. (58) found that the first hypoglycemia incidence rate was 3.70 per 1,000 patient-years (95% CI: 3.50–3.91). Rates by age were 1.77 (20–64), 3.37 (65–74), and 7.59 (≥75). Hypoglycemic events occurred more often in patients with diabetic comorbidities (58).

The precipitating factors of diabetic emergencies identified in this study reflect global trends (5, 55). A study conducted at Shanghai Tongji Hospital by Wu et al. (55) reported that the most common precipitating factors of hyperglycemic emergencies (DKA, HHS) were infections (70.3%), newly diagnosed diabetes (17.7%), and non-adherence with medication (5.7%). Similarly, a consensus report from multiple countries indicated that infection was a precipitating factor for DKA, with rates ranging from 14.0% to 58.3% across countries (5). Specifically, infection contributed to 28.6% of DKA cases in Australia, 25% in Brazil, 39.2% in China, 58.3% in Indonesia, 25.3% in South Korea, 32.5% in Nigeria, 33.2% in Spain, 47.8% in Syria, 31.7% in Taiwan, 44.6% in the UK, and 14.0–16.0% in the US (5). Furthermore, Umpierrez et al. (5) reported that the new-onset diabetes was a precipitating factor for DKA (3.3% to 23.8%), with new-onset diabetes accounting for 5.7% of DKA cases in Australia, 12.2% in Brazil, 3.3% in Indonesia, 12.8% in Spain, 18.2% in Taiwan, 6.1% in the UK, and 17.2–23.8% in the US.

Evidence from high-income settings also indicates that disengagement from routine diabetes care, including missed appointments and poor medication adherence, is strongly associated with acute deterioration in glycemic control (59–61). In a large US managed-care cohort of more than 84,000 adults with diabetes, patients who missed over 30% of scheduled visits had HbA1c levels that were 0.7% to 0.8% higher, along with poorer medication refill adherence and worse self-management compared to those who attended all appointments (59). Similarly, a systematic review of adults with T2DM found that 12% to 36% missed regular clinic visits, and that missed appointments were associated with 24% to 64% higher odds of poor glycemic control and a 60% increase in re-hospitalization (61). Large observational studies from OECD countries across Europe, America, and the Asia-Pacific region likewise reported that low adherence to chronic cardiometabolic medications more than doubled the risk of hospitalization among people with diabetes, while better adherence was linked to fewer emergency visits and hospital admissions (60).

The clinical outcomes of diabetic emergencies in Africa appear less favorable than those reported in high-income countries such as Australia, Switzerland, the UK and the US, where mortality rates from DKA and HHS are generally low (8, 62, 63). In the USA, national surveillance data showed that in-hospital case fatality for DKA declined from 1.1% in 2000 to 0.4% in 2014, despite an increase in the number of admissions (62). Similarly, in Victoria, Australia, the adult DKA mortality rate was 0.51% between 2002 and 2016, with rates of 0.69% among individuals with T1DM and 0.24% among those with T2DM (63). In the UK, a study by Gibb et al. (64) found that mortality during hospitalization for DKA did not exceed 0.16% (64). However, individuals who experienced recurrent DKA episodes demonstrated significantly higher long-term mortality rates (64). Those with a single DKA episode had a 5.2% risk of death over an average follow-up period of 4.1 years, whereas patients admitted multiple times for DKA exhibited a 23.4% risk of death over approximately 2.4 years of follow-up (64). Data from Switzerland also indicated relatively low mortality, with Ebrahimi et al. (8) reporting an adult in-hospital mortality rate of approximately 3.9% in a large DKA cohort. The mean hospital stay for older adults was about 11 to 13 days, while mortality rates remained much lower among children.

Diabetes prevalence has risen most sharply in low- and middle-income countries (4), whereas advances in treatment—and the resulting low mortality rates from diabetes-related complications—have been concentrated in high-income countries (5, 8, 62, 63), thereby widening the global treatment gap. This review shows that diabetic emergencies in Africa reflect limited resources and continuing challenges in diabetes prevention and care. Moreover, the poor clinical outcomes, including high mortality rates, chronic complications, and recurrent admissions, align with evidence indicating that delayed or inadequate management correlates with adverse outcomes (65–68). Thus, the burden outlined in this review highlights the need for prompt emergency treatment alongside stronger preventive and follow-up strategies in African diabetes care. These strategies need to align with current clinical guidelines that advocate rapid metabolic stabilization and prompt treatment of precipitating causes, particularly infections (5, 69, 70). Beyond acute management, established guidelines emphasize structured diabetes education, insulin access, glucose monitoring, and follow-up support as vital for preventing recurrence (71).

## 5 Limitations

This scoping review has several limitations related to methodology and the underlying evidence. Most of the included studies were retrospective and cross-sectional, making them susceptible to selection, recall, and information biases, which could distort estimates of the burden and determinants of diabetic emergencies. Ethiopia contributed the majority of studies, while other African regions were underrepresented, limiting the generalizability of the findings across the continent. Additionally, significant heterogeneity in study designs, case definitions, populations, and outcome reporting prevented the generation of pooled prevalence estimates, restricting the ability to provide a single summary measure of the burden of DKA, HHS, and severe hypoglycemia throughout Africa. Most studies were conducted in hospitals, particularly at tertiary facilities, with limited data from primary care or community settings, and relatively few focused on pediatric populations. This likely leads to an underestimation of milder or pre-hospital events and a lack of representation of children and adolescents. Finally, the emphasis on published, predominantly English-language literature raises the potential for publication and language bias, as relevant grey literature and non-English reports—especially from Francophone countries—may have been overlooked.

## 6 Implications of the study

This scoping review highlights that DKA was the most common diabetic emergency in Africa, followed by HHS and severe hypoglycemia. These conditions are often triggered by factors such as poor glycemic control, infections, non-adherence to treatment, and limited access to chronic care. These findings suggest that many crises may be preventable through improved primary care rather than relying solely on emergency responses. Health systems should implement integrated models focusing on routine monitoring, affordable supplies, infection screening, and patient education to address socioeconomic vulnerabilities, including rural residency and lack of insurance. Policymakers need to invest in pan-African surveillance and registries, particularly given Ethiopia’s prominence in the evidence base, to optimize resource allocation and develop effective guidelines. Researchers should use these findings to conduct prospective multi-country trials targeting modifiable risks, such as non-adherence and delayed care, and to develop context-specific tools, including community health workers and mHealth solutions, while also conducting a meta-analysis to pool prevalence estimates.

## 7 Conclusion

This scoping review indicates that diabetic emergencies constitute a significant and largely preventable source of morbidity and mortality among people with diabetes in Africa. DKA is the most frequently reported emergency, followed by HHS and severe hypoglycemia. These events typically occur in contexts characterized by poor glycemic control, infections, treatment non-adherence, irregular follow-up, and limited access to ongoing diabetes care, highlighting the interplay between health system and socioeconomic factors and individual clinical risks. Variability in study designs and reporting methods precluded pooled prevalence estimates; however, reported ranges and high in-hospital mortality rates—up to 30% in certain settings—alongside prolonged hospital stays and recurrent episodes, underscore the urgent need to enhance both primary and specialized diabetes services. Improving access to affordable insulin and glucose monitoring and integrating structured patient education and infection prevention into routine care are critical. The concentration of evidence from a limited number of countries reveals significant geographic gaps, underscoring the need for coordinated surveillance, standardized reporting, and future meta-analyses to guide targeted interventions in resource-constrained health systems across Africa.
